# Plasma adsorption in refractory chronic gouty arthritis flare: A case report

**DOI:** 10.3389/fimmu.2022.1045982

**Published:** 2022-11-24

**Authors:** Yan Lei, Tian-Jiao Cui, Xiao-Hua Wang, Xue-Mei Zhang, Chun Tang, Zhi-Hua Zheng

**Affiliations:** Department of Nephrology, Center of Nephrology and Urology, The Seventh Affiliated Hospital, Sun Yat-sen University, Shenzhen, China

**Keywords:** case report, cytokines, gout, plasma adsorption, refractory gouty arthropathy, arthritis

## Abstract

**Background:**

Along with uric acid, which is the primary driving factor of gout, downstream inflammatory mediators have been shown to be involved in the pathogenesis of gouty arthritis flares. Extracorporeal haemadsorption is an emerging technology for the treatment of dysregulated inflammatory states by effectively removing cytokines from the bloodstream. Whether haemadsorption was effective in refractory gout flares has not been reported in the literature.

**Case summary:**

We report the case of a 52-year-old male who presented with refractory gouty arthropathy for 30 years. His uric acid levels were poorly controlled due to poor diet and treatment compliance. Tophi were found to have precipitated in multiple joints and subcutaneous tissue. In the last 2 years, his incidents of gouty flares had become more frequent, and resistant to the medications, including colchicine, allopurinol, febuxostat, glucocorticoids, and NSAID analgesics. He had experienced a triad of chills, high fever and arthritis for the past 2 weeks. Therefore, he took 2 mg colchicine twice daily for 2 weeks with no improvement in his pain. Proinflammatory cytokines, such as interleukin-6 (IL-6) and tumour necrosis factor-α (TNF-α), were found to be remarkably elevated. Given that conventional treatment was unsuccessful, we tried to employ plasma adsorption (PA) to remove inflammatory cytokines. After 4 sessions, symptoms, such as fever, joint swelling and pain, were greatly improved. Meanwhile, the levels of proinflammatory factors such as IL-6 and TNF-α were found to be decreased, while the anti-inflammatory factor IL-10 remained the same during the course. He was followed up for 8 months and arthritis have flared up twice in response to a high-purine diet.

**Conclusion:**

Our study suggests that plasma adsorption (PA) may be a promising and feasible treatment for refractory gout when conventional treatments are unsatisfactory or contraindicated. However, more clinical trials are needed to verify the efficacy and safety of the treatment.

**Core tip:**

Chronic gouty arthritis flares are refractory to conventional treatment, such as uric acid-lowering drugs and NSAID analgesics. Due to the involvement of inflammatory cytokines, plasma adsorption was employed to alleviate flares by removing inflammatory mediators. Herein, we report a 52-year-old male who presented with refractory gouty arthropathy for 30 years, manifested with a triad of chills, high fever and arthritis. He underwent several sessions of plasma adsorption, and his symptoms soon improved, along with a drop in inflammatory mediators. We conclude that plasma adsorption may be a promising and feasible treatment for refractory gout when conventional treatments are unsatisfactory or contraindicated.

## Introduction

Gout is a common cause of inflammatory arthritis, and studies of its worldwide epidemiology suggest that the incidence and prevalence in both the developed and developing world are increasing (1). A typical manifestation of gout begins with an acute onset of first metatarsophalangeal joint synovitis. This is associated with nighttime attacks and is followed by severe joint pain peaking within 24 hours. These symptoms are accompanied by redness, dysfunction of the joints and fever. Tophi deposits affect limb shape and can erode bones, joints, tendons, ligaments and skin, causing joint deformities, dysfunction, nerve compression, and skin ulcers. Apart from uric acid, which is the primary driving factor of gout, downstream inflammatory mediators have been shown to be involved in the pathogenesis of gout flares or gouty arthritis, such as IL-1, TNF-α, IL-6, and IL-8. An IL-1 inhibitor, canakinumab, has been approved for the treatment of refractory gout in European Union (2). Extracorporeal cytokine haemadsorption is an emerging technology that is being used for the treatment of dysregulated inflammatory states by effectively removing cytokines from the bloodstream (3). Therefore, in this case, as we report below, we employed plasma adsorption in an attempt to treat a patient with frequently relapsing gout flares and gouty arthritis that who was resistant to conventional therapy.

## Case presentation

### Chief complaints

A 52-year-old male patient was admitted to the Seventh Affiliated Hospital of Sun Yat-sen University with a triad of chills, fever and arthritis for 2 weeks.

### History of the present illness

The patient had refractory gouty arthropathy for 30 years. He had been prescribed many uric acid-lowering drugs and analgesics, such as colchicine, allopurinol, febuxostat, glucocorticoids, and NSAID analgesics, to alleviate his complaints. The uric acid level was poorly controlled due to poor diet and treatment compliance. Tophi were found to have precipitated in multiple joints and in subcutaneous tissue. In the last 2 years, his incidents of gouty flares had become more frequent (about once or twice a month), and resistant to medications, including colchicine, allopurinol, febuxostat, glucocorticoids, and NSAID analgesics. Repeated eruptions of “pus” (likely monosodium urate crystal, MSU) were drained from his toes. Two weeks prior, he had developed a triad of chills, high fever and arthritis (mainly involving interphalangeal joints, elbow joints, and wrist joints). Therefore, he took 2 mg colchicine twice daily for 2 weeks with no improvement in his pain.

### History of past illness

The patient had a history of ischaemic heart disease for 8 years, and was diagnosed with chronic kidney disease 5 years earlier. He denied medical history of hypertension and diabetes, without habit of drinking and smoking.

### Physical examination

**Figure 1:**
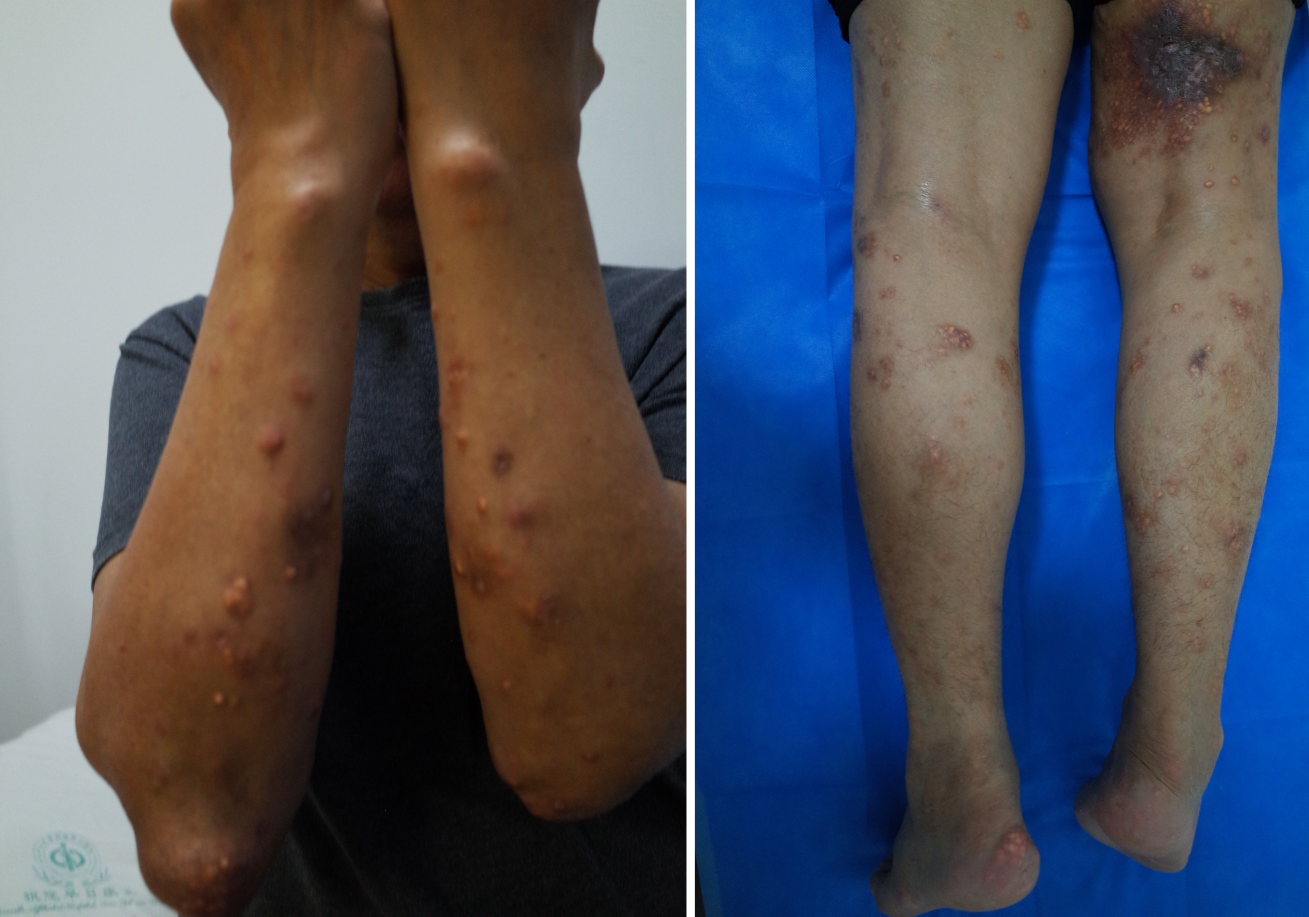
Tophi were found to have precipitated in the subcutaneous tissue and skin ulcerations were observed. The subcutaneous tophi deposits were similar to papules on the forearms, shins, and inner thighs and contained a chalk-like substance. Subcutaneous gout on the thigh also resulted in skin ulceration.

On hospitalization, the patient was alert with a blood pressure of 98/66 mm Hg, heart rate 100 beats/min, respiratory rate 18/min, and body temperature 38.2°C. His height was 168 cm, body weight 75 kg, and body mass index 26.6 kg/m^2^. Tophaceous deposits were found in subcutaneous tissues and multiple joints (**Figure 1**), which had led to the development of articular deformities. He had lost the fine motor function of his fingers. Scars were found scattered on his toes because of repeated cycles of “pus” eruptions followed by wound healing. We assessed that the joint pain score was 8–9 points by the visual analogue scale (VAS, with a dynamic range from 0 to 10 points).

### Laboratory examinations

His laboratory results revealed a normal serum UA level of 403 µmol/L (reference range 210–420 μmol/L) (owing to the irregular administration of febuxostat), creatinine at 117 µmol/L (reference range: 57–97 μmol/L), estimated glomerular filtration rate (eGFR) of 74 mL per minute, elevated white blood cell counts (WBC) (13.93*10^9/L, reference range 3.5-9.5*10^9/L), neutrophil counts (12.1*10^9/L, reference range 1.8-6.3*10^9/L), C-reactive protein (CRP) (21.4 mg/L, reference range<10 mg/L) and erythrocyte sedimentation rate (ESR) (40 mm/h, reference range 0-15 mm/h), and a normal procalcitonin level (PCT) (<0.05 ng/mL, reference range <0.05 ng/mL).

### Imaging examinations

None.

### Final diagnosis

Gout flare.

### Treatment

**Figure 2:**
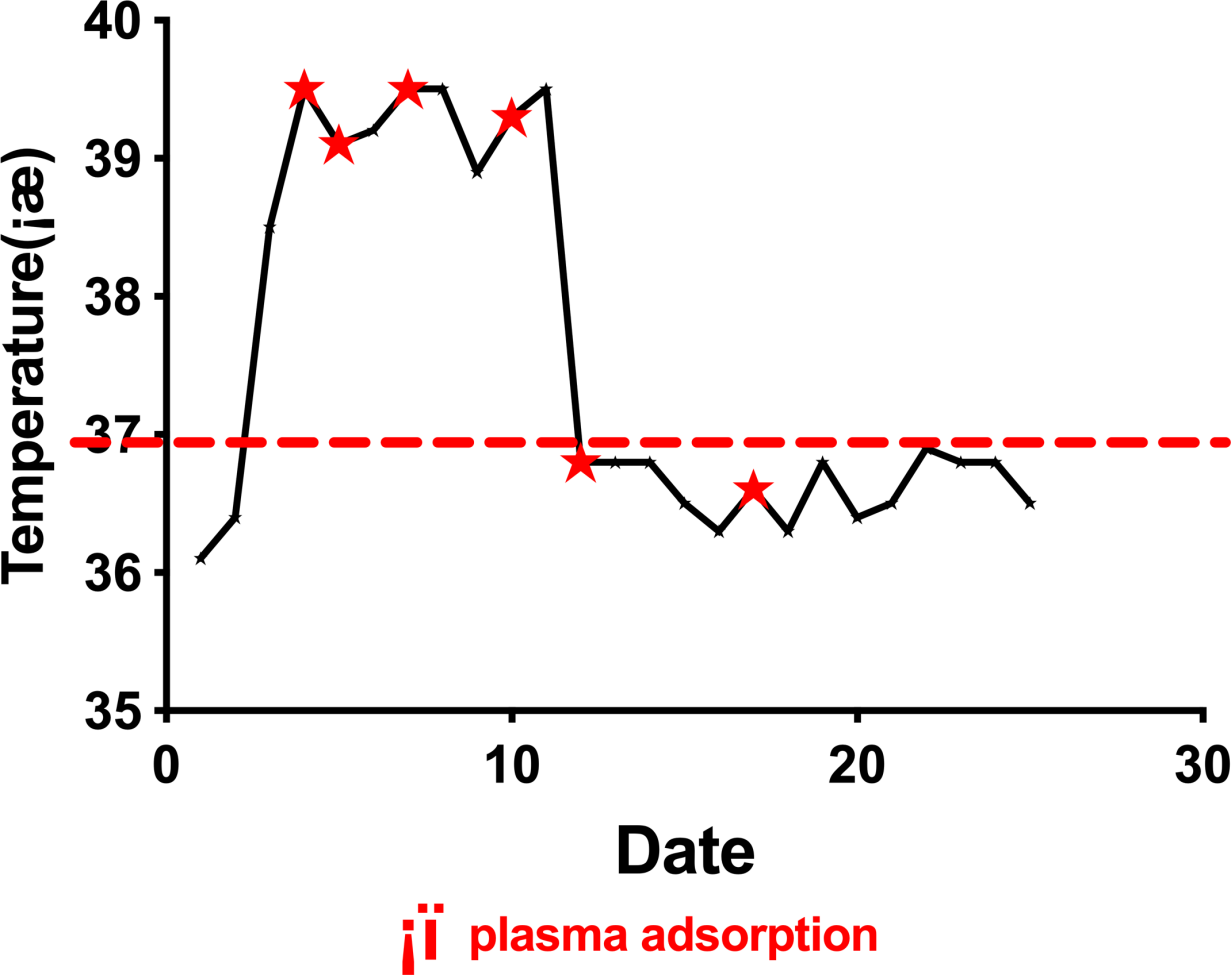
Fluctuations in body temperature during treatment. 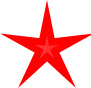
plasma adsorption.

Febuxostat, glucocorticoids, and NSAID analgesics failed to control the symptoms, although serum UA levels were found to have lowered to normal levels after conventional treatment (331.6 µmol/L). On the 4^th^ day of hospitalization, the patient began to experience chills followed by high fever (T_max_ 39.5°C) (**Figure 2**) and arthralgia once again. Blood tests showed increased white blood cell and neutrophil counts (16.08*10^9/L and 13.91*10^9/L respectively), as well as elevated PCT levels (0.27ng/mL). Repeated blood cultures were found to be negative. This was accompanied by extremely elevated IL-6 and TNF-α levels of 184 pg/mL (reference range <8.1 pg/mL) and 53.9 pg/mL (reference range <5.9 pg/mL), respectively.

Routine drug therapy had limited effects, probably due to the existing cytokine storm. Then, blood purification was employed to remove inflammatory cytokines and excess colchicine. We performed PA (Multifiltrate CiCa ^®^, FMC, Bad Homburg, Germany; plasma filter: P2, Fresenius Germany; sorbents: Mediosorb, Bellco, Italy) every two to three days along with enoxaparin sodium to prevent coagulation after obtaining written informed consent.

### Outcome and follow-up

**Figure 3:**
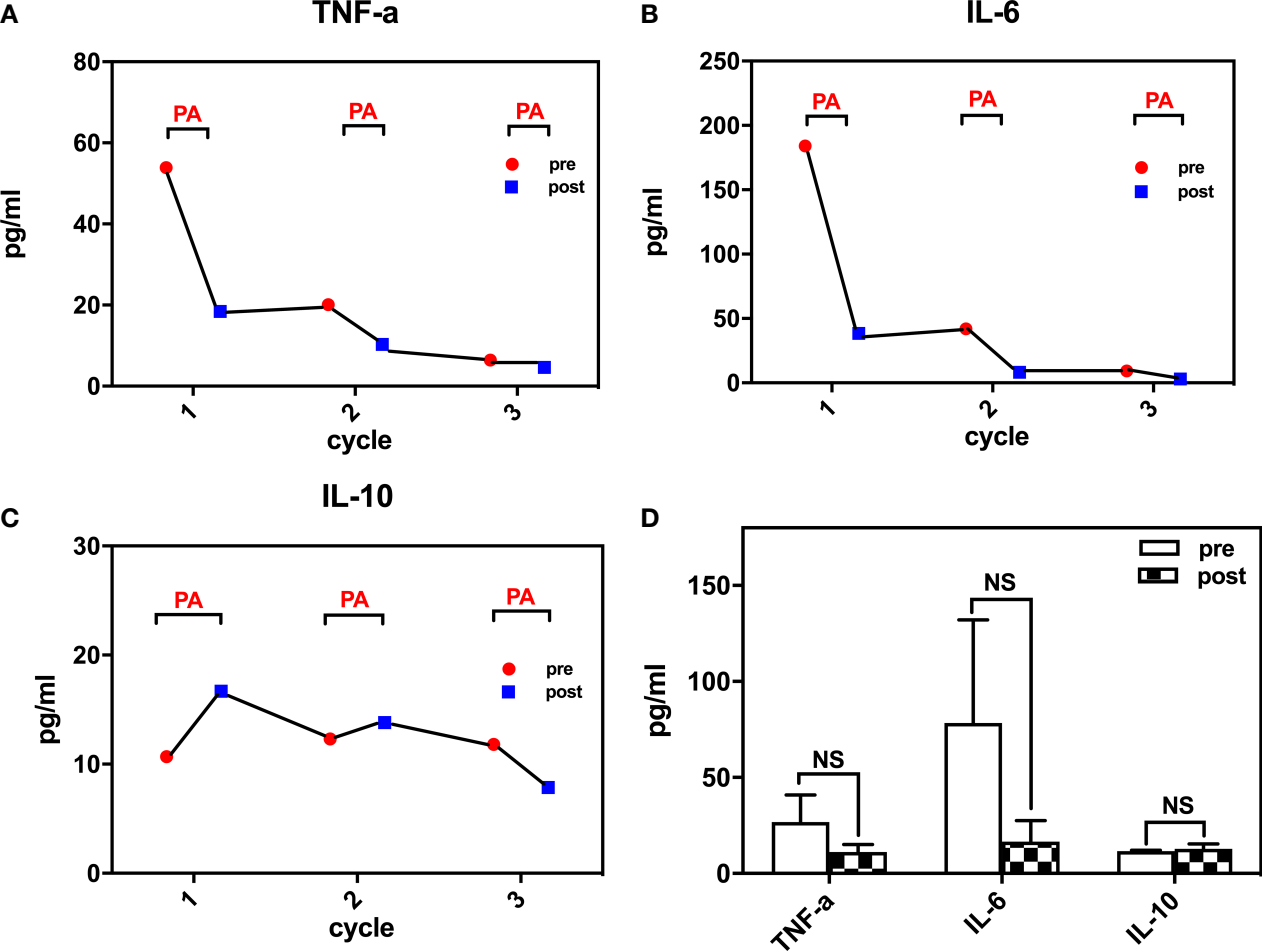
Inflammatory cytokines changes during treatment **(A)** TNF-a levels during the course; **(B)** IL-6 levels during the course; **(C)** IL-10 levels during the course; **(D)** changes of TNF-a, IL-6, IL-10 pre- and post- plasma adsorption). TNF-a, tumor necrosis factor-a; IL-6, interleukin-6; IL-10, interleukin-10. NS, not significant.

After 2 cycles of therapy, proinflammatory cytokines such as IL-6 and TNF-α levels declined, but symptoms did not improve as expected. Although he had moderate pain relief, there were large fluctuations in body temperature, which were accompanied by an exacerbation of swelling and pain in his extremities for approximately 2 days after treatment. After 4 treatment sessions, the patient had become afebrile, and his symptoms had improved significantly with reductions in swelling and joint pain. During the 5 cycles of plasma adsorption therapy, proinflammatory cytokines such as IL-6 and TNF-α were found to continue declining until they reached normal levels, whereas the levels of the anti-inflammatory cytokine IL-10 eventually stabilized (**Figures 3A–C**). This was accompanied by notable improvements in the clinical manifestations of gouty arthritis. Finally, there were 6 treatment sessions in total. However, due to the sharp fluctuation in the values and the small sample size, we did not find any significant difference in IL-6 and TNF-α before and after PA treatment (**Figure 3D**). No adverse events were found for the treatment. During follow-ups over the next 8 months, the patient’s tophi was stable and gouty arthritis was found to only have flared up twice in response to a high-purine diet, with no fever reported and a joint pain score of 4–5 points (VAS). These symptoms were alleviated by NSAIDs, and the patient’s serum uric acid was found to be maintained at an acceptable level over the long term.

## Discussion

Gout is a common metabolic disease caused by elevated levels of uric acid, which leads to the deposition of monosodium urate crystals in the joints and kidneys. The inflammatory response to monosodium urate crystals mediated by macrophages and neutrophils leads to acute gout, which can develop into chronic tophaceous gout (CTG) (4). CTG usually manifests as recurrent gout attacks. However, the treatment of CTG is typically challenging. The activation of the Nod-like receptor pyrin domain-containing protein 3 (NLRP3) inflammasome leads to the release of IL-1β and other proinflammatory cytokines that are essential to gout pathogenesis (5). IL-1 inhibitors, like canakinumab (a specific recombinant human antihuman IL-1β monoclonal antibody) and anakinra (IL-1 receptor antagonist) were not commercially available in our country. Extracorporeal cytokine haemadsorption is an emerging technology that is being used for the treatment of dysregulated inflammatory states. Plasma adsorption (PA) has been found to remove various proinflammatory cytokines *in vitro* and in animal models of sepsis (6). With the rapid development of adsorbents, PA has been widely used in sepsis (7, 8), hyperlipidemia (9) and autoimmune diseases (10, 11). Previous studies demonstrated PA could decrease mortality rate of critically patients (7, 8) and favor the treatment efficacy (9, 10). The duration of treatment depends on saturation time of adsorbents, generally according to manufacturer’s recommendations, ranging from 3-6 hours to 24 hours. The frequency of PA is performed based on the doctor’s judgement of patients’ condition, ranging from every day to 1-3 times a week. Evaluation of therapy effect is based on the different indications (7–11). The adverse effects of PA may include hypotension, arrhythmia, allergic reaction, infection, embolism, hemorrhage, hemolysis, and rupture of the plasma separator membrane, but with fairly rare occurrence. In this report, we used PA to treat a case of gout flare and an acute attack of CTG when conventional drugs were unable to control the attack. The duration was based on the instructions of adsorbents. With reference to autoimmune diseases (9, 10), PA frequency was set at every two to three days. And PA treatment course was defined as at least three therapeutic sessions or according to the doctor’s judgment. We planed in advance to finish PA treatment when the signs, symptoms and labratory examinations of patient were improved.

Notably, during the first two sessions, the patient’s symptoms did not improve as expected, even though IL-6 and TNF-a levels decreased considerably. Only after the next 2 treatment sessions were the patient’s symptoms greatly improved. We speculated that inflammatory factors may be released temporarily from the joints or other local tissues after these factors are eliminated from the circulatory system during plasma adsorption. When a state of balance was reached, inflammatory factors then started decreasing to a lower level both within the tissue and in the blood. This led to a termination of the cytokine storm, which was then accompanied by a dramatic relief of symptoms, such as chills, fever and the associated joint swelling and pain.

## Conclusion

Based on our analysis of the available literature, this is the first time that plasma adsorption has been applied to relieve gout flares and chronic tophaceous gout. We believe that the improvement and relief of symptoms after such treatment can be partially attributed to a rebalancing of anti-inflammatory and proinflammatory cytokines in the body. This study suggests that plasma adsorption may be a promising and feasible treatment for refractory gout when conventional treatments are unsatisfactory or contraindicated. However, more clinical studies are needed to confirm the therapeutic role of hemoadsorption in refractory gout episodes.

## Data availability statement

The raw data supporting the conclusions of this article will be made available by the authors, without undue reservation.

## Ethics statement

The studies involving human participants were reviewed and approved by Ethics committee of seventh Affiliated Hospital of Sun Yat - sen University. The patients/participants provided their written informed consent to participate in this study. Written informed consent was obtained from the individual(s) for the publication of any potentially identifiable images or data included in this article.

## Author contributions

YL and T-JC wrote the manuscript and performed follow-ups with the patient. X-HW, CT and Z-HZ revised the manuscript. X-MZ collected data. All authors have read and approve the final manuscript. All authors contributed to the article and approved the submitted version.

## Funding

Supported by the Shenzhen Science and Technology Innovation Committee of Guangdong Province of China (Grant No. JCYJ20180307150634856, JCYJ20210324123200003)

## Acknowledgments

We would like to give special thanks to Yunsheng Xu from the Seventh Affiliated Hospital of Sun Yat-sen University for language support. We also thank the nurses and physicians for their care of this patient.

## Conflict of interest

The authors declare that the research was conducted in the absence of any commercial or financial relationships that could be construed as a potential conflict of interest.

## Publisher’s note

All claims expressed in this article are solely those of the authors and do not necessarily represent those of their affiliated organizations, or those of the publisher, the editors and the reviewers. Any product that may be evaluated in this article, or claim that may be made by its manufacturer, is not guaranteed or endorsed by the publisher.
